# Exploring the Laryngectomy Pathway Experience—a Mixed‐Method, Patient Journey Mapping Study of Two US Health Centres

**DOI:** 10.1111/1460-6984.70326

**Published:** 2026-09-02

**Authors:** Laurie Wennerholm, Clare L. Burns, Elizabeth C. Ward, Jasmine Foley, JK Rasamny, Craig Berzofsky, Gina Palma

**Affiliations:** ^1^ School of Health and Rehabilitation Sciences The University of Queensland Brisbane Queensland Australia; ^2^ Department of Speech‐Language Pathology – Perlmutter Cancer Center and Rusk Rehabilitation NYU Langone Health New York New York USA; ^3^ Speech Pathology Department The Royal Brisbane and Women's Hospital, Metro North Health Queensland Health Brisbane Australia; ^4^ Centre of Functioning and Health Research, Metro South Health, Queensland Health Brisbane Australia; ^5^ Department of Otolaryngology, Head and Neck Surgery White Plains Hospital White Plains New York USA; ^6^ Department of Otorhinolaryngology Montefiore Medical Center Bronx New York USA

**Keywords:** care pathways, laryngectomy, patient experience, patient journey mapping, service delivery, speech‐language pathology

## Abstract

**Background:**

Consensus guidelines advocate multidisciplinary care to optimise recovery following laryngectomy. However, limited evidence exists regarding the nature of the patient recovery journey or their experience accessing rehabilitation services.

**Aims:**

This study examined the services accessed by, and experiences of, individuals with a laryngectomy (IWL) in the first 12 months post‐surgery.

**Methods and Procedures:**

Patient journey mapping methodology was used to explore the healthcare journey and patient experience of 12 IWL across the first 12 months of rehabilitation. Demographics and detailed service data were collated from medical records and later reviewed for accuracy with each participant. Individual interviews were used to explore issues that influenced care experiences, and participants also provided experiential ratings relating to the emotional impact of each appointment (positive/negative/neutral) and the reason for the rating. Journeys were explored collectively as a group, then 3 detailed patient journeys are presented highlighting similarities and differences in participant experiences.

**Outcomes and Results:**

Participants attended a median (Mdn) of 7 medical visits (range = 4–14), 12 allied health visits (range = 4–45), saw 13 different professionals (range = 6–22), and travelled to 6 locations (range = 2–6) for healthcare. Factors adding to the complexity of the health care journeys included preparation for surgery, nature of surgery (primary versus salvage), access to trained specialists throughout phases of care, coordinating between services, travel and funding for consumables. Despite issues, participants largely rated experiences as positive (Mdn = 77; range = 37%–100% positive). Participant perspectives on issues impacting their healthcare journey formed 6 themes: (1) Preparation for laryngectomy surgery, (2) Recovery post‐surgery (3) Access to skilled and coordinated services, (4) Approach of healthcare staff, (5) Communication difficulties impacting healthcare, and (6) Accessing laryngectomy resources and costs.

**Conclusions and Implications:**

Following laryngectomy, a wide range of multidisciplinary services are accessed to support recovery and rehabilitation. Examining patient experiences through journey mapping has highlighted challenges that can influence the care pathway. Addressing issues that impact service access and patient experience will assist in enhancing laryngectomy care pathways and improve the patient experience.

**WHAT THIS PAPER ADDS:**

*What is already known on the subject*

- Current care pathways advocate specialist, multidisciplinary care for optimal recovery for individuals with a laryngectomy (IWL). Although existing laryngectomy guidelines specify that a range of specialist services are required, and detail what those services should entail, what this actually looks like and means for IWL as they live this path of recovery, remains largely unexplored.

*What this study adds to the existing knowledge*

- This work expands the body of evidence in laryngectomy care by exploring the healthcare journeys of a cohort of IWL to better understand the extent of medical and allied health services required in the first 12‐months post‐surgery and the patients’ experiences of that care. This work is the first to use journey mapping, a methodology that provides detailed insights gained from both quantitative and qualitative data to elucidate the patients’ care experience. Understanding the patients lived experience, and any challenges faced, is essential information needed to continue to inform future care optimisation.

*What are the clinical implications of this study?*

- The collective findings from this study highlight the potential challenges IWL can face accessing care in the post‐acute phase. Participants attended a high number of medical and allied health visits, from a variety of professions and services, and faced several challenges including being prepared for their recovery, accessing skilled professionals, communication with healthcare staff, and costs of necessary consumables/equipment. Healthcare providers, including speech‐language pathologists, must recognise and address these issues where possible, to optimise care and reduce patient burden.

## 1 Introduction

Laryngectomy is one of the most radical surgeries of the head and neck region, creating a multitude of complex and permanent physical, functional, and psychosocial consequences (Hinni and Crujido 2013; Ward et al. 2007). To optimise recovery and rehabilitation, patients require support from a highly skilled multidisciplinary team (MDT) (Dawson et al. 2025; Messing et al. 2019), and international cancer service documents recommend that this specialist care should be integrated across the patient care continuum from diagnosis to long‐term survivorship (Cancer Council Victoria and Department of Health Victoria 2021; Homer et al. 2024; NCCN 2023; Verdonck‐de Leeuw et al. 2022). However, the process of integrating care across several healthcare services and professionals is inherently challenging.

Therefore, to maximise service efficiency, optimise the patient experience, and limit patient and caregiver burden, it is recommended that head and neck cancer (HNC) care be provided through an integrated care pathway (Chen et al. 2000; Messing et al. 2019). Care pathways provide a ‘road map’ for professionals, patients, and caregivers to help navigate the healthcare process (Messing et al. 2019). They help to standardise care to help avoid variation from patient to patient (Middleton et al. 2001). Although there is currently limited data on laryngectomy care pathways reported in the literature (Chen et al. 2000; Hanna et al. 1999, Levin et al. 2000), evidence suggests that receiving early acute post‐surgical care within a coordinated pathway benefits both individuals with a laryngectomy (IWL) and the services providing care (Hanna et al. 1999; Levin et al. 2000).

While such studies have demonstrated the value in using care pathways in the early phases of laryngectomy management, there has been little exploration of the nature of the patient care journey, or pathway of care, beyond the acute hospital stay. At present, cancer service guidelines only broadly describe the healthcare services accessed for post‐discharge laryngectomy care (NCCN 2023). At minimum, IWL are recommended to attend ongoing reviews with a head and neck surgeon, oncology team and specialty trained nurses for disease surveillance and medical treatment (i.e., wound care, medication management, etc.). A variety of allied health services are also recognized as vital for comprehensive care, including speech‐language pathologists (SLPs) to facilitate communication and swallowing rehabilitation which are key to restoring quality of life (Clarke et al. 2016; Dawson et al. 2025; DeMaddelena 2002); Longobardi et al. 2023), registered dietitians (RDs) for nutritional management, and social workers (SWs) and psychologists for psychological wellbeing and support regarding lifestyle adjustments (Licitra et al. 2016). It has also been noted that IWL may need to travel to multiple facilities to access all these various healthcare services (Massa et al. 2019).

Although it is currently unknown exactly what accessing care looks like for IWL in the post‐acute phase, what is needed to manage their rehabilitation needs or the patient experience of this stage of their care, existing research has confirmed that issues do exist in post‐surgical care. A recent study revealed that patients experienced moderate levels of distress, especially in the early post‐surgical phase, and perceived substantial gaps in their care including inadequate preparation for the extensive physical, functional and socioemotional changes resulting from laryngectomy (Chapman et al. 2026). Other work has shown there are substantial differences with the educational materials and provider preparedness between different settings supporting IWL, highlighting potential variability in care received depending on service provider (Watson et al. 2025). While research exploring the issues of females who undergo laryngectomy surgery has highlighted a lack of adequate supports for female patients with regards to reintegration into society, changed family roles, intimacy, cosmesis and their unique altered sense of self (van Sluis et al. 2020).

Considering that the nature and patient experience of the post‐acute care pathway for IWL is largely unknown, the objective of the current study was to build an understanding of this phase of care by exploring the individual care experiences of a cohort of IWL using patient journey mapping. Patient journey mapping offers a methodology to examine and document the health care journey, highlighting the patient's perspective (Meyer 2019; Trebble et al. 2010). Through creating a visual representation of the path ‘walked’ by the patient, journey mapping allows deficiencies and strengths in healthcare pathways/processes to be identified (Kushniruk et al. 2020), which in turn can be used to inform the streamlining of healthcare practices in complex clinical populations (Alkandari et al. 2019; Kelly et al. 2017; Kushniruk et al. 2020; Ly et al. 2021). It also involves collecting insights into the patient's experience. Typically involving individual (Meyer 2019) or small numbers of participants (Bulto et al. 2024; Foley et al. 2022; Saragosa et al. 2023), journey mapping incorporates both quantitative and qualitative data to enable in‐depth, rich descriptions of the patient's lived experience, rather than taking a health service perspective (Bulto et al. 2024; Foley et al. 2022; Saragosa et al. 2023).

This study is the first of a series of studies examining the current care pathway for IWL, its strengths as well as obstacles, to inform future development of an evidenced based care framework for SLP management of this complex clinical population. These findings will provide insights into both nature and types of healthcare services accessed as part of recovery and rehabilitation post laryngectomy, and potentially identify factors that patients perceive are assisting, or challenging healthcare access for themselves and their family. Also considering the vital role of the SLP in pre‐operative and ongoing education and support, post‐operative care and long‐term management of IWL for communication, swallowing and pulmonary rehabilitation (DeMaddelena et al. 2002; Longobardi et al. 2023), it is hoped that these learnings will help enhance current SLP service pathways for IWL.

## 2 Method

### 2.1 Design

Journey mapping involved collection of detailed, mixed‐methods data for a cohort of patients post laryngectomy who had received their laryngectomy surgery from two different services in the United States (US) health system. These two sites were part of a large health system and were deliberately chosen to ensure the study captured participant experiences associated with different service models. Site 1 was a 290‐bed suburban community hospital with a separate, but co‐located, cancer centre. Site 2 was a 750‐bed, urban, tertiary, academic medical centre with onsite outpatient cancer services. Both facilities had an MDT HNC team incorporating head and neck surgeons (HN surgeons), plastic surgeon/reconstructive specialists (PS), SLPs, medical oncologists, radiation oncologists, RDs, physical therapists (PTs) and SWs. Site 1 had been providing HNC services for only 5 years and managed a growing volume of patients, however many medical appointments were conducted offsite, 20 miles or more from Site 1. Site 2, the larger institution, had been providing comprehensive HNC care for over 15 years, and offered all appointments on‐site. In both services, referral to SLP services was initiated by the diagnosing, lead physician/medical practitioner at the time of diagnosis/planning for laryngectomy surgery. Need for referral to other services required during the post‐acute period could be initiated by any member of the MDT or the patient, with written referral to these other services authorized by the lead physician in primary care of the patient at the time (i.e., HN surgeon, radiation oncologist).

This research was conducted with full ethical approval from relevant institutional review boards including White Plains Hospital (WPH), Montefiore Medical Center (MMC)/Albert Einstein School of Medicine (WPH, MMC Ref # 061125, IRB# #2020‐11238) and The University of Queensland (UQ # 2020001304/ʼʼ#20‐030). Reporting of the qualitative components of this study were guided by the COnsolidated criteria for REporting Qualitative research (COREQ) Checklist (Tong et al. 2007).

### 2.2 Participants

Eligible patients had undergone a laryngectomy (± partial or circumferential removal of the pharynx) as primary or salvage treatment for cancer, had a viable method of communication including surgical voice restoration, writing, text‐to‐speech, or electrolaryngeal use, attended SLP services at one of the participating sites, and were at least 12 months post‐laryngectomy surgery. Participants also had to be able to independently consent, communicate effectively via their alaryngeal mode of communication, and have no history of cognitive impairment that may impact on their ability to participate in an interview. Patients with recurrent disease or on palliative treatment were excluded. Potential participants who met the study criteria were informed of the study via a flyer provided to them by the administration officer when they attended their next SLP consult. Those interested in participating were then provided with the information and consent forms. All participants provided written informed consent.

### 2.3 Procedures/Data Collection

Demographic data collected included gender, age, diagnosis (Tumour Node Metastasis (TNM) stage), any prior HNC treatment, date of laryngectomy, employment, social status including the presence of a caregiver, and the location of home residence. Data about services included hospital admissions, outpatient appointments incorporating—date, location, return distance travelled from home to appointment, health services accessed and professional(s) seen, health complications relating to laryngectomy care, and any other procedures relating to laryngectomy care. Difficulty accessing medical records at Site 1 resulted in some missing data and this is clarified in the results. Service data for each participant were then listed chronologically to map the services accessed for their laryngectomy care over the 12‐month period.

Study data were collected in two parts. Part 1 involved collection of demographic information, then details of the healthcare appointments relevant only to laryngectomy care attended in the first 12 months post‐surgery as recorded in the electronic medical record (EMR). Where appointments could not be confirmed by the EMR (i.e., for services provided in other facilities), these were verified by patient report later in Part 2 of this study. Details of any appointments or medical services accessed for any other medical care or management of co‐morbidities unrelated to their laryngectomy were not collected.

In Part 2, both in‐person interviews and the collection of experiential ratings were used to capture the patient experience (Meyer 2019: Swann 2017). Part 2 was conducted approximately 6 months after the participants were first consented due to the time taken to collect the Part 1 data, and additional delays created by the COVID‐19 pandemic. Individual semi‐structured interviews (15–30 min duration) were conducted at the facility by the treating SLP working in the laryngectomy caseload (LW, GP—Master degree level) at each hospital site. Both interviewers were female and had clinical relationships with participants. Both were clinicians working within the services, had over 18 years of experience working with IWL, and approached this study with an understanding of the content of the research and issues that patients can experience. As it was anticipated that having had a clinical relationship with the interviewer may have influenced participants responses about services received, all participants were explicitly told that the study was being conducted to understand the current care pathway experienced after laryngectomy as part of LW's PhD research, and they were encouraged to speak freely about both positive and negative aspects of their past experience. At the participant's discretion, a caregiver was able to be present in the interview for support. Two participants chose to have a caregiver present.

The interview prompts were created by EW (PhD level researcher with extensive experience in interviewing and qualitative research in HNC) and LW (PhD student). During these interviews the IWL: (1) reviewed the data collected regarding their laryngectomy care appointments across the 12 months post‐surgery and identified any relevant appointments not documented on the map, (2) discussed their overall experience of attending the health services related to their laryngectomy care in an interview format (see interview guide—Appendix), and (3) rated the emotional impact of each appointment during their laryngectomy care experience by labelling it with an experiential descriptor: positive (+), negative (‐) or neutral (N) and explained the reason for the rating. Throughout all data collection, the lead investigator (LW) maintained field notes to capture observations, interactions with participants and any other key contextual information. They also maintained reflexive diary notes about the dynamics of the interviews and if there were any concerns about power‐imbalance, or personal factors that could have potentially influenced reporting.

### 2.4 Analysis

The service data for all participants were reviewed and key patterns explored across the cohort. This included collating the number of inpatient admissions (acute care or rehabilitation facilities); number of medical appointments attended, and cancer treatment visits (chaemotherapy, radiotherapy or hydration); number of allied health and clinical nurse navigator appointments attended; number of professions accessed; and number of different professionals involved in care, including the number of different SLPs involved. Of note, the nurse navigator was only present at Site 1‐ employed as a breast cancer navigator and was involved with only very complex cases to facilitate referrals to other sub‐specialists (i.e., referral to a head and neck lymphoedema specialist). Distance required to travel to appointments from the participants’ residential address to the health facility was calculated via Google maps and expressed as a round trip, in miles. Recognising there can be issues with recollecting services received in the past, the time from each patient's surgery to them attending the Part 2 stage to review and discuss their journey was reported in months. Quantitative data were analysed descriptively expressed as median, mode and range (rounded to the nearest whole number).

Patients’ experiential ratings were analysed descriptively and expressed as a percentage of the appointments attended. Interview data were audio‐recorded and analysed using qualitative content analysis with an inductive approach (Graneheim and Lundman 2004) by two members of the research team (CB, LW). Transcripts were read multiple times to become immersed in the data and minor issues with intelligibility were discussed by the two researchers and resolved. Transcripts of the interviews were maintained by the study team, they were not returned to the participants at any time. Key responses or phrases about challenges experienced were then extracted from the data and assigned codes. These codes were then grouped into broad categories and discussed and revised until consensus was reached. Representative quotes were extracted that aligned to patient experiences. Although the sample size of the interviewed cohort was small, no new themes were identified and established themes were reinforced in later interviews suggesting data saturation. The potential sufficiency of the sample can also be considered consistent with the emerging contemporary model of ‘information power’ (Malterud et al. 2016), whereby the more salient and information‐rich the sample, the fewer participants are required.

Finally, visual patient journey maps that represented all key information about demographics and services accessed across the 12 months of laryngectomy care were created for three selected individuals. These three individuals were chosen to demonstrate the care pathway of a patient who (a) received a primary laryngectomy within the tertiary care service, (b) received a primary laryngectomy within the suburban service, and (c) had a salvage laryngectomy, to highlight different experiences. The methodology for presenting the data for these individuals followed the journey mapping process described by Meyer (2019).

## 3 Results

All eligible patients who were approached consented to participate. Initially there were 18 participants recruited, 10 from Site 1 and 8 from Site 2. This represented 50%–60% of the IWL currently being managed by SLP services at each site. Three consenting participants were later removed due to incomplete medical data (i.e., large number of services that occurred in other public/private facilities that were unable to be accessed) and 3 others were later excluded for other reasons (involvement in a medicolegal case, illness) leaving 12 eligible participants (9 from Site 1, 3 from Site 2) (∼45% of the total laryngectomy population from Site 1 and ∼12% of the total from Site 2). Of the 12 IWL, data were collected for all 12 individuals in Part 1, however, only 9 completed Part 2, as 3 had deceased between the time of consent and prior to completing the interview and experiential ratings. It is acknowledged that more participants were lost to attrition from Site 2. However, as the intention of including 2 sites was purely to capture diversity in the care pathways experienced by participants, not to compare care models between services, this had little impact on the final analysis.

Demographic data of the 12 individuals are displayed in Table 1. Due to the small sample size and specific characteristics of the cohort, demographics have been reported in a summary format, as recommended by Braun and Clark (2019) to avoid potential risk of identifying participants. The sample included 12 individuals recruited from 2 care centres; the majority of participants were from Site 1. The cohort was predominately male with advanced disease. Participants primarily had squamous cell carcinoma, with one patient having verrucous type. Subsites included transglottis (*n* = 5), glottis (*n* = 4), supraglottis (*n* = 2) and one patient (*n* = 1) had a primary supraglottic cancer and a second primary of the oropharynx/base of tongue. Over half of patients received laryngectomy surgery in a salvage context while the remaining participants underwent primary surgery followed by adjuvant chaemoradiotherapy. All patients underwent surgical voice restoration with the majority receiving secondary voice prosthesis placement. Patients travelled an average of 24 miles round trip from their homes to healthcare appointments. The average time from laryngectomy surgery to patient interviews about their care experiences was 27 months. Of note, half the participants (50%) had a caregiver and less than half (46%) were employed pre‐surgery.

**TABLE 1:** Whole cohort characteristics (*N* = 12).

Characteristic	Summary
Sites	Two centres (Site 1: *n* = 9; Site 2: *n* = 3)
Age (years)	Range: 43–79 (mean age = 63 years)
Gender	Male: *n* = 10; Female: *n* = 2
HNC stage	Predominantly advanced disease (majority T3–T4; with nodal involvement present in some cases, no distant metastasis)
Tumour type	Primarily squamous cell carcinoma; 1 verrucous carcinoma
Surgical indication	Salvage: *n* = 7; Primary: *n* = 5
Adjuvant treatment	CRT: *n* = 5; None: *n* = 7
Timing of TEP	Secondary: *n* = 9; Primary: *n* = 3
Travel distance	Approximately 4–52 miles (mean distance = 24 miles)
Time from surgery to interview	Range: 12–41 months (mean time = 27 months)

Abbreviations: CRT, chaemoradiotherapy; HNC, head and neck cancer; T, tumour; TEP, tracheoesophageal puncture.

### 3.1 Care Experiences

Health care services accessed by the 12 participants are displayed in Table 2. In the first 12‐months following surgery, participants had a median (Mdn) of 3 inpatient admissions (range 1–4), attended a Mdn of 7 medical visits (range 4–14), and a Mdn of 12 allied health appointments (range 4–45). There was a paucity of clinical/nurse navigator visits as neither site had a designated navigator for HNC care. Those patients who received adjuvant chaemoradiotherapy (*n* = 5) attended the required 33–42 outpatient treatments.

**TABLE 2:** Service access and experiential ratings for the 12 participants.

		Inpatient	Outpatient visits				Multidisciplinary professionals				
Participants	Site	No. inpatient admissions	No. medical visits	No. cancer treatment appointments RT/CRT	No. allied health visits	No. navigator /nurse visits	No. professionals involved in care	No. professions	No. SLP services accessed	No. of locations accessed for care	Experiential ratings
1^a^	1	4	6	0	7	0	17	13	3	6	44% positive 4% negative 52% neutral
2^a^, ^b^	1	3	6	0	45	1	22	13	4	6	37% positive 4% negative 59% neutral
3	1	3	4	37	12	0	12	10	2	5	69% positive 14% negative 17% neutral
4^a^	1	2	14	0	10	1	13	11	3	6	81% positive 0% negative 19% neutral
5^a^	1	3	12	0	4	0	20	12	3	6	66% positive 6% negative 28% neutral
6^a^	1	4	11	0	16	0	18	11	4	6	Deceased
7	1	3	11	35	7	0	13	10	2	6	Deceased
8^a^	1	2	5	0	7	0	6	6	1	4	100% positive 0% negative 0% neutral
9^b^	1	4	8	42	19	0	11	10	2	4	77% positive 0% negative 23% neutral
10^a^	2	4	6	0	12	0	15	11	2	3	100% positive 0% negative 0% neutral
11	2	1	7	38	11	0	9	9	1	2	Deceased
12^b^	2	2	9	33	17	0	8	8	1	2	100% positive 0% negative 0% neutral
Analysis											
*Median*	—	3	8	0	12	0	13	11	2	6	77% positive 0% negative 19% neutral
*Mode*	—	4	6	0	7	0	13	10	1	6	100% positive 0% negative 0% neutral
*Range*	—	1–4	4–14	0–42	4–45	0–1	6–22	6–13	1–4	2–6	37%–100%positive 0%–14% negative 0%–59% neutral

^a^ Denotes salvage surgery

^b^ Denotes patient with a journey map in results section.

Across the group, a median of 13 different professions provided care, ranging from 6 to 22 individual clinicians. Most participants accessed care from more than 10 different professions. Specific to SLP services, patients attended a Mdn of 2 different services (range 1–4) during the 12‐month post‐operative period.

Participants travelled between 2 and 6 sites (Table 2) to receive care. Participants from Site 1, the less established HNC service, travelled to between 4 and 6 locations for care, while participants from Site 2, although still accessing a high number of professionals, travelled to only 2–3 locations to receive their care. Overall, patients travelled between 4 and 52 miles (round trip) to attend appointments, with a Mdn distance of 24 miles travelled per appointment. Exploring the individual data from the 12 participants (Table 2), it was noted that for patients who underwent salvage surgery (denoted by superscript a in Table 2), the greater complexity of their condition often necessitated a wider range of services, greater numbers of professions involved in care, and many travelled extensive distances to access care (Table 2).

Collective analysis of the group's experiential ratings about the services they received is also displayed in Table 2. This revealed largely positive care experiences (range 37%–100%) with negative and neutral experience ratings ranging from 0%–14% and 0%–59%, respectively. The median values for experiential ratings were 77% (positive), 0% (negative) and 19% (neutral). Across the individual data, it was however noted that participants who underwent a salvage laryngectomy rated their healthcare experiences slightly less positively than those who had primary surgery.

Qualitative analysis specifically focusing on the challenges raised in the participant interviews revealed six themes. These six themes, their subcategories and an exemplar quote for each theme are detailed in Table 3. Additional quotes have also been embedded here within the text to provide further context. Theme 1, ‘Preparation for Surgery’, describes perceived inconsistency reported by patients with respect to the adequacy of information provided by the healthcare service and access to a laryngectomy support visitor, to help prepare them for surgery and functional changes following laryngectomy (e.g., Site 1, Participant 3‐*‘What's gonna be on the other end of my surgery…they never told me I would wake up with 300 staples up and down my arm… not being able to speak’*). In ‘Recovery Post‐surgery’ (Theme 2), patients reported distress when identifying medical complications and how to manage these successfully (e.g., Site 1, Participant 3 – *‘Because I kept leaking (fistula)… and once he (physician) taught me to pack it, it was a lot of self‐care…’*). The importance of access to psychological counselling was highlighted as critical to support adjustment post‐surgery as well (see Table 3). In Theme 3, participants discussed the challenges ‘Accessing Skilled and Coordinated Services’, with some highlighting a lack of primary care staff (i.e., those who provide first contact, general medical care, and provide referral to specialists, including physicians, nurses, etc.) with laryngectomy skills or coordination of overall care. Theme 4, ‘Approach of Healthcare Staff’, captured the variable interpersonal rapport and/or lack of individualised care that was provided by some healthcare staff (e.g., Site 1, Participant 1 – *‘She was a very good doctor., but she is busy and doesn't give patients that individual care… I needed that’*). In Theme 5 ‘Communication Difficulties Impacting Healthcare’, participants had variable success/ease in making appointments and booking transportation for follow‐up care. Many reported challenges in communicating with health care staff due to difficulties talking on the phone with no alternative communication option offered and needing to rely on others for assistance (e.g., Site 2, Participant 11 – *‘When I first got this (surgery), I couldn't even talk, but then they came up with this (TEP) and I love it’*). Finally, in Theme 6 ‘Accessing Laryngectomy Resources and Costs’ accessing and paying for laryngectomy supplies was a common problem. Participants reported reliance on healthcare professionals to order supplies and confusion around the funding schemes for consumables. Some found insurance companies would not fund key consumables, while others were required to cover out‐of‐pocket expenses (e.g., Site 1, Participant 1 ‐ *‘Luckily I was able to control the 20% (uncovered by insurance). I can control out of pocket expenses when it comes to prescriptions and supplies*’).

**TABLE 3:** Six themes pertaining to challenges experienced during the healthcare journey.

Categories	Subcategories	Example participant quotes
1. Preparation for surgery	Receiving adequate information	“…. there are things you could have done differently to prepare people for what they go through”. (Site 2, P10)
	Access to peer support visitor	“It would've been helpful to talk to a laryngectomy patient”. (Site 1, P1)
2. Recovery post‐surgery	Managing medical complications	“I was concerned because all of a sudden now it's like, yellow, yeah, God its infected. And I'm going… Is this normal?” (Site 1, P8)
	Counselling/psychological care	“I can see how maybe a counsellor would've helped people, but not me”. (Site 2, P12)
	Awareness of concomitant conditions	“I didn't realise that I my GERD would make my voice sound bad…As long as my stomach is ok, my voice is ok”. (Site 2, P10)
3. Access to skilled and coordinated services	Lack of primary care staff with laryngectomy skills	“I got sick a couple of times and my regular doctor couldn't help me. I came to Dr. [HN surgeon] and he helped me”. (Site 2, P10)
4. Approach of healthcare staff	Variable interpersonal rapport/individualised care	“The hospital personnel who don't seem to have the patience, and they are not willing to go through with you and hear you and all that and it can be kind of frustrating” (Site 1, P1)
	Support from SLP	“I have you (SLP). Who is a very big support in my life. Without you and your encouragement I wouldn't be here today. I would have given up…But I don't think anybody would take the time especially with me….in the beginning I was a rough case”. (Site 1, P3)
	Healthcare providers' role unclear	“I had in‐home nursing… all she did was take my temperature, take my vitals and leave? I could do this by itself. Yeah. Why do I need you? Right?” (Site 1, P3)
5. Communication difficulties impacting health care	Appointment making	“I couldn't make phone calls—can't communicate on the phone”. (Site 1, P2)
	Contacting health professionals	“My primary care physician I can't reach him by email…You should be able to access everything by email”. (Site 1, P1)
	Booking travel to healthcare appointments	“Transportation was a problem in the beginning…I really couldn't speak well. …So to schedule my transportation my sister did it all for me. So I felt kind of helpless”. (Site 1, P3)
6. Accessing and paying for laryngectomy supplies	Accessing initial supplies	“Getting the supplies I needed in the beginning was a little hard… but it worked out”. (Site 2, P12)
	Superfluous supplies	“I was sent [home] with a lot of stuff. But I didn't use it at all”. (Site 1, P4)
	Monitoring supplies by healthcare staff	“The visiting nurse…when she came, she would check the supplies, and she would order whatever we needed.” (Site 1, P4)
	Lack of understanding of funding models to access supplies	'Within two days, we just said no (to home health care), because if we use you (SLP), then we can get the supplies. That part was very confusing to me… but maybe because it's Medicare, and Medicare doesn't help at all'. (Site 1, P8)
	Insurance companies not supporting costs	'It was challenging to pay for HMEs. Insurance doesn't want to cover it'. (Site 1, P9)
	Need to cover out‐of‐pocket expenses	'Luckily, I am able to control the 20%. A decision where I can control out of pocket expenses, when it comes to prescriptions and supplies'. (Site 1, P1)

Abbreviations: GERD, gastroesophageal reflux disease; HMEs, heat moisture exchangers; SLP, speech‐language pathologist.

### 3.2 Individual Journey Maps

**FIGURE 1:**
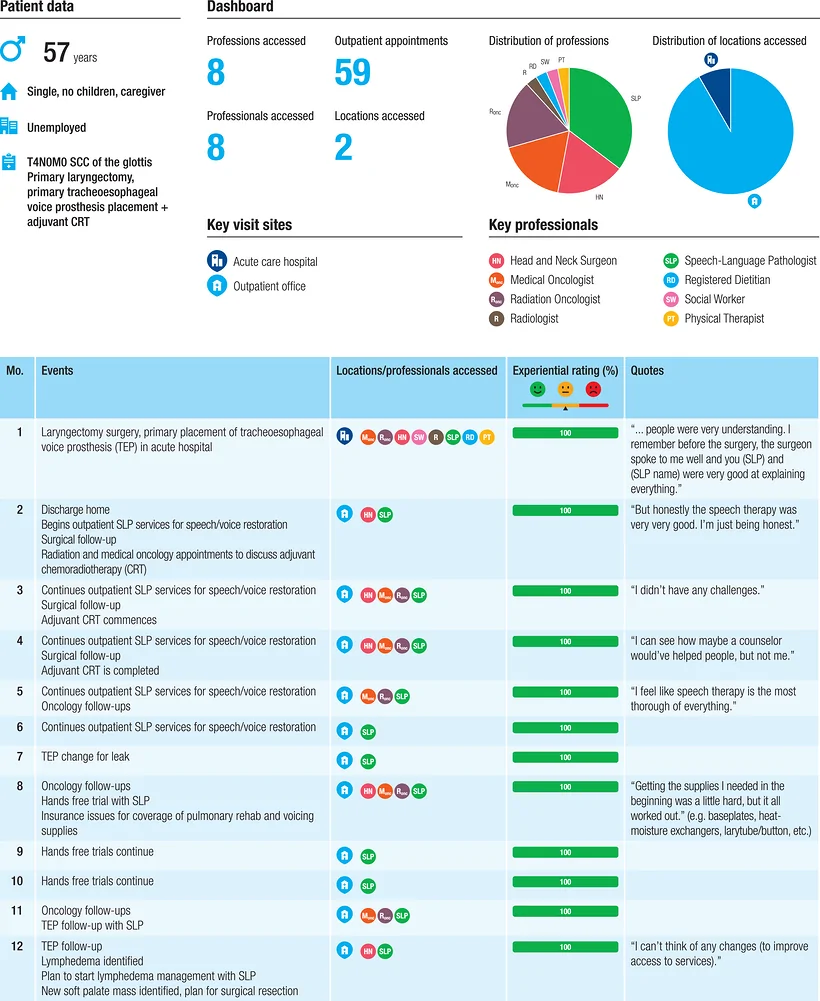
Participant 12 Journey Map.

**FIGURE 2:**
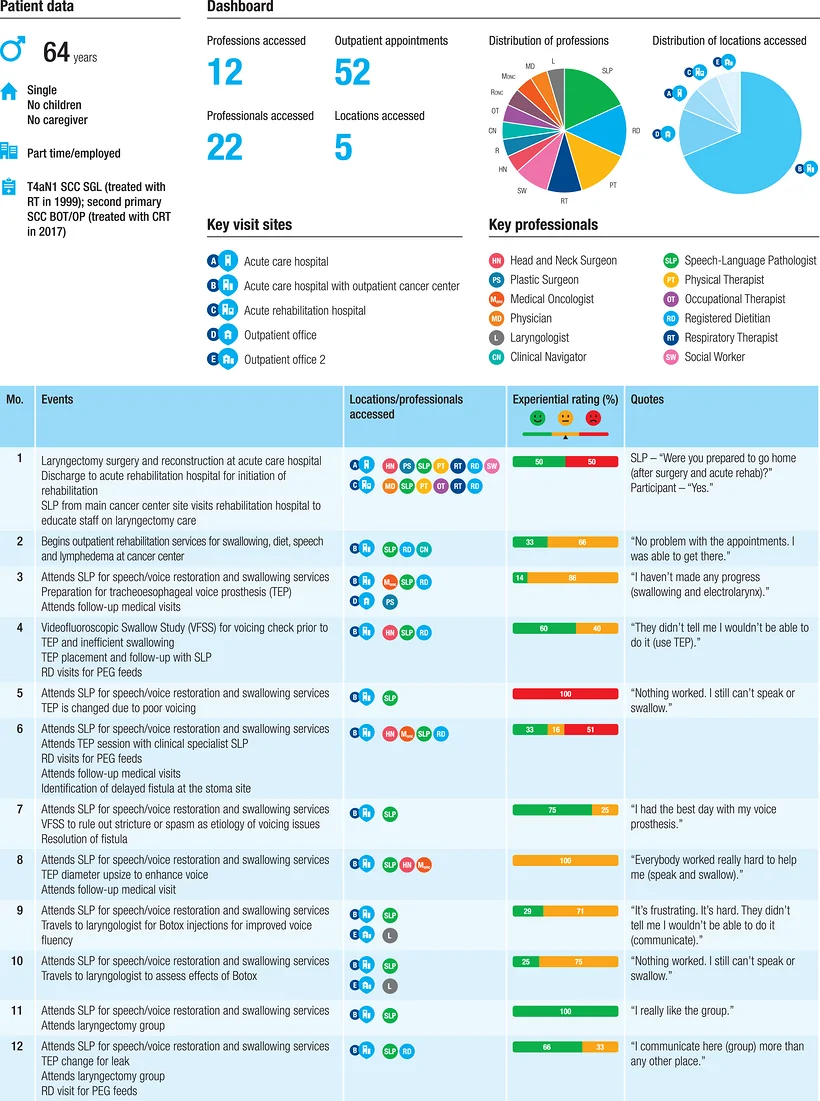
Participant 2 Journey Map

**FIGURE 3:**
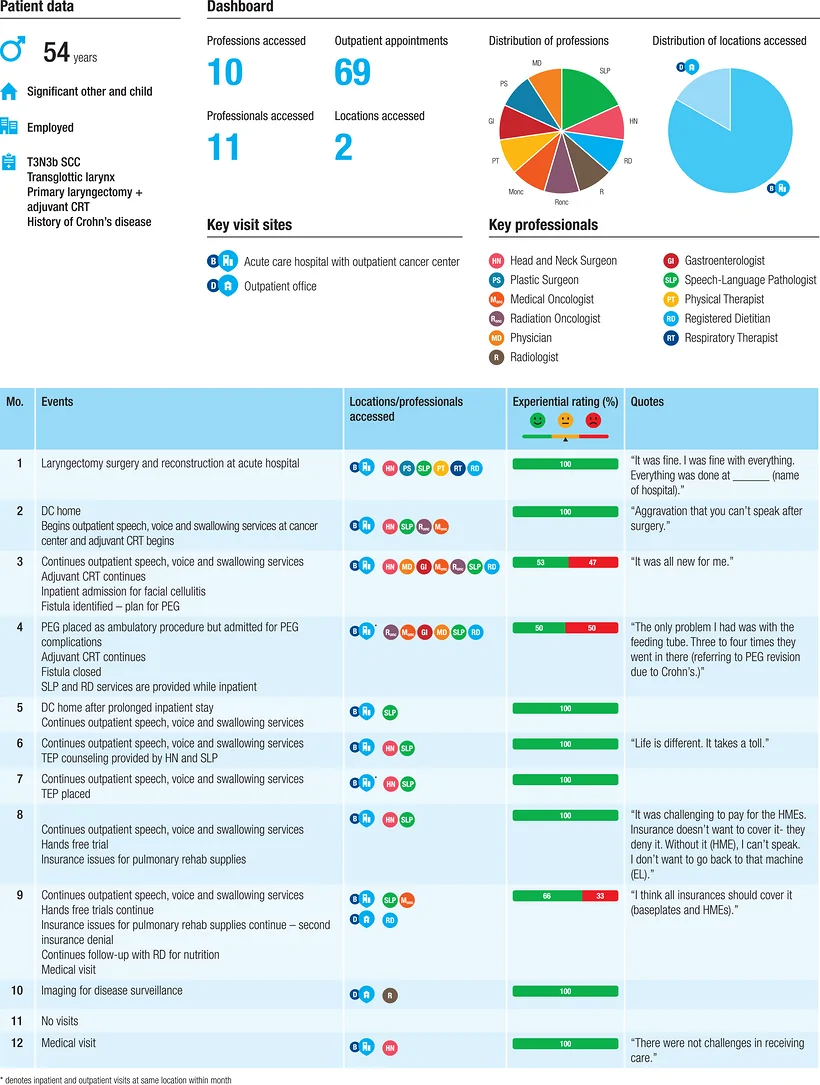
Participant 9 Journey Map

Although the quantitative care experience data and the qualitative data in Table 3 provide a summary of challenges experienced across the participant group, at an individual level there was naturally variability in the nature and extent of issues faced by individuals in their own care path. To illustrate this individuality, 3 detailed journey maps (Figures 1, 2, 3) are provided as examples of individual patient experiences. These journey maps detail the individual participant, their engagement with services and key service events, and provide experiential ratings and quotes relating to positive/negative perceptions of these key stages in the journey. How each individual engaged with services, and did, or did not, experience care challenges is detailed in the following summaries.

#### 3.2.1 Patient Journey Map 1: Participant 12

Participant 12 is an independent, unemployed single 57‐year‐old male with no children (Figure 1). He was admitted to hospital (Site 2) with progressive otalgia, stridor, dyspnoea and dysphonia, and diagnosed with T4N0M0 glottic SCC in 2019. He was assessed by head and neck surgery, radiation oncology and medical oncology and due to the extent of his tumour, a total laryngectomy was recommended with primary tracheoesophageal puncture (TEP) and voice prosthesis placement. Pre‐operatively he attended education and counselling by the specialist SLP. Post‐operatively, he communicated effectively with an electrolarynx prior to commencing trials using the voice prosthesis. During his acute hospitalisation, he also received care from the RD, PT and social worker.

Post discharge he accessed outpatient services (Site 2). He attended regular SLP sessions and communicated effectively using the voice prosthesis. Early central leakage due to stricture was managed with insertion of a specialised voice prosthesis. At 4‐months post operatively, he completed a course of CRT. During this treatment he received coordinated care from his healthcare team including the radiation oncologist, medical oncologist and head and neck surgeon.

As outlined by the positive experience ratings and nature of the quotes contained in Figure 1, Participant 12 faced few of the challenges outlined in Table 3 and expressed largely positive experiences with his care journey. He experienced a relatively uncomplicated medical course. However, in regard to overall care burden, it must still be noted that within the first 12 months this participant attended 59 outpatient appointments with 8 different professions. Although care was accessed at 2 locations, the majority was provided at a single facility located only 4 miles (approximately 20‐minute drive) from his home (Table 1) and he expressed no problem with accessing skilled services or attending appointments (Theme 3, Table 3), stating—*‘It was never hard to access the services I needed’*. His main challenge was obtaining and paying for equipment/consumables for his laryngectomy management (Theme 6) *‘getting the supplies I needed in the beginning was a little hard’*. It was only after advocacy by the healthcare team and multiple discussions with the insurance company, he reported that *‘it (obtaining supplies) all worked out’*. He also alluded to adjustment/psychological issues with laryngectomy, suggesting that a mental health counsellor could have been helpful for some— *‘but not me’*.

#### 3.2.2 Patient Journey Map 2: Participant 2

Participant 2 is a 64‐year‐old male (Figure 2). He was single and worked full‐time. In 1999, he was diagnosed with T4aN1M0 supraglottic SCC which was treated with radiotherapy. Eighteen years later, he presented with a T4aN0M0, p16+ left tongue base SCC which was treated with CRT. A percutaneous endoscopic gastrostomy (PEG) was placed prophylactically. Several months after treatment, he developed chondronecrosis of the larynx, and a salvage laryngectomy was performed at an external hospital, as at the time, Site 1 was not equipped to manage the complex reconstruction. During his inpatient stay, Participant 2 accessed services from 7 different professions.

During recovery, Participant 2's communication and self‐care were limited, necessitating a 1‐month stay an acute rehabilitation facility. The SLP, nurses and RT were not experienced in laryngectomy care at this site, therefore, the SLP and HN surgeon from Site 1 provided clinical education and advice to these staff. During this period, Participant 2 accessed care from 6 different specialties including SLP, PT, OT, RD and RT.

After discharge home, Participant 2 attended intensive speech, swallowing and lymphoedema therapy at the co‐located cancer centre (Site 1). He underwent a secondary TEP for voice prosthesis placement, and for 10 months, he attended 1–2 SLP appointments per week for communication and swallowing rehabilitation. He attended follow‐up appointments on‐site with 6 different specialists including: RD, PT, radiation oncologist, medical oncologist, HN surgeon and a clinical navigator, who supported care coordination. He also attended 2 appointments with a plastic surgeon off‐site.

Participant 2's course of voice and swallowing rehabilitation following his salvage surgery was one of the more complicated journeys of this study's cohort. Despite attending intensive SLP services and troubleshooting TEP management with an external clinician specialist SLP, tracheoesophageal voice production remained minimal. A swallow study revealed possible pharyngoesophageal segment spasm, and he attended 4 appointments with a laryngologist off‐site for Botox injections; however, ultimately voice restoration was unsuccessful. Due to progressive radiation fibrosis and cranial nerve neuropathies, electrolarynx use was also unsuccessful and Participant 2 remained reliant on writing/text to speech devices for communication. Similarly, despite intensive SLP treatment, his swallowing function only marginally improved. He chose to forgo oral food/fluid and rely on PEG feeds. He transitioned from 1:1 SLP sessions to the weekly laryngectomy group meeting (Site 1), for communication practice and peer support. He reported enjoying the group and provided support to other patients.

Consistent with the complexity of his recovery, within the 1‐year post‐laryngectomy he had accessed 22 professionals from 13 professions, incorporating 6 medical and 45 allied health visits. In contrast to Participant 12 above, Participant 2 accessed care from 5 different locations, which ranged from 5–20 miles (approximately 30‐minute drive) from his home location (Table 1). Although he was accessing a high number of different services, he reported no issues physically accessing services ‘*[I had] no problem with the appointments. I was able to get there’*.

Unlike Participant 12, Participant 2 rated most of his care experiences as neutral (59%), another 4% as negative, with just a third (37%) being positive (Table 2). One of the key negative issues experienced related to what was perceived as a lack of adequate preparation for surgery (Theme 1, Table 3) particularly regarding voice restoration ‘*They didn't tell me I wouldn't be able to do it (use a TEP successfully)’*. His course of recovery and managing the medical complications post‐surgery was also challenging (Theme 2, Table 3). In particular, his communication recovery was slow and frustrating *‘I haven't made any progress’*. His slow communication recovery also added to issues accessing services. Amongst other things, frustrations arose particularly when making appointments (Theme 5, Table 3) *‘I couldn't make phone calls—can't communicate on the phone’*. He rated many of his SLP visits as neutral and when probed about the reason for this, he stated, *‘Everyone worked really hard to help me… Nothing worked. I still can't speak or swallow’*. Although unable to return to full time employment, he was offered only sporadic work with a *‘kind’* employer *‘who feels bad for me’*.

#### 3.2.3 Patient Journey Map 3: Participant 9

Participant 9 is a 54‐year‐old male with Crohn's disease who was diagnosed with T3N3bM0 transglottic SCC (Figure 3). He attended pre‐operative counselling by the SLP and underwent a primary laryngopharyngectomy with reconstruction at Site 1. During his inpatient stay, he saw a core team of 5 professionals comprising a HN surgeon, plastic surgeon, SLP, PT and RD. His recovery was uneventful, and he was discharged home 10 days post‐surgery, managing full oral intake and communicating effectively using an electrolarynx.

Post‐discharge he attended outpatient appointments at Site 1 with the SLP, HN surgeon, medical and radiation oncologist. He commenced CRT and attended weekly SLP appointments for communication and swallowing rehabilitation. He was followed closely by the RD, to monitor his Crohn's disease during CRT. Complications arose in the first month of treatment, when he was admitted to hospital (Site 1) for 2 days for treatment of facial cellulitis and then developed a pharyngocutaneous fistula. PEG placement was recommended, however severe stomach scarring secondary to Crohn's disease caused inherent complications. He was re‐admitted (Site 1) for 1‐month where the gastroenterology service attempted multiple PEG revisions. Communication with the HNC team was suboptimal and once the PEG was placed, the fistula had closed, and he was discharged home on an oral diet.

Following CRT, Participant 9 attended weekly outpatient visits at Site 1 with the SLP and RD. He underwent a secondary TEP and voice prothesis placement and made additional visits to the SLP to obtain laryngectomy consumables as his insurance did not cover all the equipment he required. He expressed difficulties with insurance coverage for essential voice and pulmonary rehabilitation equipment. He attended several visits in conjunction with an external SLP consultant, to become proficient using a hands‐free voicing system. He also attended appointments with the on‐site HN surgeon, medical oncologist and radiation oncologist, and off‐site plastic surgeon via telehealth.

Like most participants in this study, multiple professionals were involved in this patient's care, (11 professionals from 10 different professions) in the first year following laryngectomy. Similar to Participant 12, the majority of Participant 9's cancer services were provided at one site (Site 1), which was located 16 miles (approximately a 25‐minute drive) from his home (Table 1). His overall experiential rating of care was positive (77%) with no visits rated negatively, and 23% rated neutrally (Table 2). He reported no issues with accessing staff or services stating *‘It was fine. Everything was done at (Site 1)’* and *‘there were no challenges in receiving care’*. Key challenges that were discussed, however, related to Themes 4 and 6 (Table 3). Regarding the approach of healthcare staff (Theme 4), this participant reported issues with the lack of patience of healthcare staff, stating, ‘*some of them (nurses) treat the patients like they nobody. Some of them won't understand you or they don't want to be bothered’*. This participant also experienced financial issues (Theme 6) relating to poor insurance coverage and need to appeal to the insurance company to fund his healthcare equipment were noted as a key frustration. He stated, *‘It was challenging to pay for the HMEs. Insurance doesn't want to cover it—they deny them. Without it (an HME), I can't speak. I don't want to go back to that machine [an electrolarynx]’*. He was able to return to work part‐time, however the limited hours caused financial stress for this family, and he reported it was challenging to communicate with clients who lacked patience.

## 4 Discussion

Using patient journey mapping, this study has provided insights into the complexities of the care journey experienced by IWL. Collective analysis of the experiences of a range of participants from two different health centres revealed that individuals accessed a high number of allied health visits and received care from numerous healthcare professions and professionals for their rehabilitation needs during the initial 12 months post‐laryngectomy. Despite the intensity of appointments required, patients described their healthcare experiences largely as positive but also described several healthcare‐related challenges. While international cancer service frameworks advocate for coordinated multidisciplinary services across the continuum of care (Bickford et al. 2018; Cancer Council Victoria and Department of Health Victoria 2021; Homer et al. 2024; NCCN 2023), current healthcare pathways for IWL reported in the literature are limited to the immediate, post‐operative phase, and fail to describe the breadth of services accessed to support recovery and rehabilitation (Chen et al. 2000; Hanna et al. 1999; Levin et al. 2000). Through documenting the patients’ care journey and their healthcare experiences, this study has revealed findings that can be used to inform health care pathways for IWL in the rehabilitation phase of care.

A key finding of this study was the number of facilities and professionals visited by IWL across the participant group. Not surprisingly, patients accessing care from the suburban hospital with its less established MDT, were required to access care across an increased number of sites. These findings are consistent with Beeram et al. (2021) who reported greater challenges for patients with HNC treated in community‐based settings due to complex travel requirements. The literature reports several service‐related challenges arising from engaging multiple sites, including duplication of services which can incur higher healthcare costs and poorer reimbursement, as well as a potential breakdown of communication and access to patient records across facilities (Rourke 2021; van Walraven et al. 2010). There is an increased risk that care fragmentation can occur when patients receive treatment from several providers or sites. Specifically for patients with HNC, care fragmentation can result in poorer clinical outcomes, elevated patient and caregiver distress and burden (Beeram et al. 2021) and higher rates of morbidity and mortality (Chen et al. 2019; Garstka et al. 2020; Graboyes et al. 2017). Researchers have advocated solutions to streamline services such as employing a nurse navigator to coordinate care or designating a contact person from each care site to facilitate communication amongst HNC MDT and health care sites (Graboyes et al. 2023; Kagan et al. 2020). These service investments can optimise care coordination and reduce healthcare burden for IWL.

Current evidence supports that IWL should have access to clinical specialists with skill sets consistent to meet their care needs (Charlton et al. 2015, Foley et al. 2022; Hancock et al. 2020; Hinni and Crujido 2013; Ward et al. 2007); however, since laryngectomy represents a small clinical subpopulation, it remains difficult for all professionals to gain specialisation in this clinical area (Lewis et al. 2016; Maddox and Davies 2012). One of the factors influencing patients transitioning to primary care post discharge was varying staff skill mix. Multiple professionals from the same discipline were often involved in a single patient's care, necessitating a higher number of appointments, and increased travel and care burden for the patient. Varying expertise and appointments with the same profession across several facilities and in primary health care settings, are common experiences of patients living in locations distanced from cancer centres (Beeram et al. 2021; Foley et al. 2022). To address deficiencies in skill mix, several authors have identified ways to overcome access challenges. Burns et al. (2017) implemented an SLP telehealth service that shared care between a specialist cancer centre and regional sites for IWL. That model enabled patients to access care at their local facility facilitated by a specialist clinician while training the local SLP to gain competence in laryngectomy management. Foley et al. (2022) also developed a suite of resources for supporting and upskilling rural HNC SLPs providing care for patients with HNC. Strategies implemented included an online education portal, formalised telehealth handover procedures, and shared‐care clinical sessions between specialists and inexperienced clinicians via telehealth for collaborative care and mentoring. Only one participant in the current study reported attending a medical appointment via telehealth and it is unknown whether telehealth services were available for other patients to access their healthcare appointments. Due to the complexity of healthcare across this patient population, there is a need to explore and evaluate flexible models of care such as telehealth to facilitate professional upskilling and to offer greater access to specialised care for HNC patients (Ewers et al. 2023) including IWL.

Experiences of the individuals in this study also highlighted how primary versus salvage surgery further impacted complexity of care for a number of participants. Enhanced care needs for individuals undergoing salvage laryngectomy have been highlighted previously in the literature (Saade et al. 2025). Permanent tissue damage from prior CRT can result in a multitude of medical issues, such as post‐operative haematoma, wound infection, and pharyngocutaneous fistula, necessitating longer hospital stays/readmissions (Meulemans et al. 2020), and lead to long‐term functional complications (Goepfert et al. 2017). Some of the study participants who underwent a salvage laryngectomy experienced these complications and consequently had increased healthcare requirements compared to their primary surgery counterparts. Understanding the complexities that may arise and the differences in care needs is essential not only to plan healthcare resources for different patient groups, but also to counsel those IWL with greater medical complexity/co‐morbidity around potential complications and care requirements.

Despite the complex care pathways experienced by many in this study, participants rated their experiences as largely positive. This could be due to the high level of supportive care provided by healthcare personnel and the positive relationships forged between the patient and professionals, particularly the SLP, during recovery. Watson et al. (2022) examined the laryngectomy patient experience both before and after the COVID‐19 pandemic and found that patients consistently reported the value of positive relationships, including those with healthcare professionals to make them feel ‘normal' again. Dooks et al. (2012) examined the experiences of IWL as they reintegrated into society post‐surgery. A key subtheme identified in that work was the patients’ need for ‘empathetic understanding' during their journey which is congruent with the current study, where many participants expressed the need for healthcare staff to have greater patience and understanding as they navigate this life changing surgery. Similarly, an important theme that emerged from the current study's findings centred around the importance of interpersonal rapport between patient and healthcare personnel and the value of this support, particularly from the SLP. This confirms the need for healthcare providers to not only facilitate physical recovery but also support psychosocial adjustment and emotional wellbeing as critical components in the rehabilitative phase following laryngectomy.

An important and growing area of research is financial toxicity in healthcare and its negative impact on patients, their caregivers and health outcomes (Jacobson et al. 2012; Johnson et al. 2008). Patients with HNC, encompassing IWL, are particularly vulnerable to financial distress, considering they commonly represent a lower socioeconomic group, have a chronic health condition often with concomitant communication and swallowing issues, and face social stigma from coworkers with poor preparation on return to work from healthcare providers (Massa et al., 2019; Massa et al. 2022; Miller et al. 2023). Not surprisingly, up to one third are unable to return to work after treatment or work only in a limited capacity (Chen et al. 2021; Mott et al. 2022). Findings from one study indicated that almost 70% of a cohort of 73 HNC patients relied on at least one “cost coping strategy” to manage the financial burden of their care, including utilising all or most of their savings, selling possessions or property, or enlisting other family members to work additional hours to meet their care needs (de Souza et al. 2017). While the financial status of the current cohort of participants was not formally recorded, many patients in this study reported financial difficulties post‐surgery due to challenges returning to work, restricted employment opportunities, challenges getting time‐off work to attend multiple appointments, and ongoing financial burden with affording laryngectomy consumables. These findings highlight the need to embed greater financial support into healthcare models with literature suggesting the inclusion of a financial health specialist within the HNC MDT to provide advice and assistance to minimise economic burden (Beeram et al. 2021; Farrugia et al. 2021). As one aspect of the financial burden for IWL includes extensive equipment costs crucial for voice restoration and pulmonary rehabilitation, the SLP must consider this burden especially for patients with limited resources, and advocate for improved institutional, public and insurance supports (Stanisce et al. 2023).

### 4.1 Limitations

Lack of access to all healthcare records for recruited participants resulted in reduced study sample size. This issue also highlights the challenges for clinical services when multiple facilities do not have integrated healthcare records. Participant recollection of appointments may also have resulted in an underreporting of visits and may have impacted their experiential ratings/responses. Participant experiential ratings were collected by a staff member from the health service; therefore, a potential reporting bias (under reporting of negative care experiences) must be acknowledged. Patient journey mapping is a methodology that collects rich data on small patient numbers and as such, next steps would be to validate the issues raised here in larger cohorts. However, given the low incidence of laryngectomy (all types) with estimation of approximately 4500 per year in the US and an overall 30%–50% decrease in radical surgery over the past 2 decades in favour of organ preservation (Saraswat hula et al. 2023), obtaining a larger cohort for future studies will involve recruitment from multiple sites. A further limitation is that this research only reports on the complexity of laryngectomy specific care, and only for patients who underwent surgical voice restoration. It did not report on the care pathway for those patients using alternative alaryngeal communication methods (e.g. electrolarynx) which could reflect different results and may be an area for future investigation. In addition, for patients with co‐existing comorbidities who need to access additional services for the management and support of those conditions, it is expected their engagement with health services would be even more complex, potentially adding to health care burden and financial stress—another area for future research. Finally, this study was restricted to a specific group of healthcare facilities within a privatised health system, therefore results may not be representative of services provided in other regions of the USA and/or internationally, or those that operate under differing funding models (e.g. public health services). Authors suggest future research be conducted in different geographical regions and with diverse reimbursement systems. Finally, larger studies could explore whether any specific demographic variables such as gender, urban versus suburban sites, primary versus salvage laryngectomy, caregiver support, travel distance, employment status, etc. particularly influence the care experience for IWL.

## 5 Conclusion

In this patient sample, journey mapping has helped to illustrate the complexity of the post‐acute care pathway for IWL in the first 12 months post‐surgery. Participants accessed care from a multitude of professionals within a number of specialty professions. Although IWL rated their health care experiences as largely positive, they also described several challenges faced with respect to the phases of care, their healthcare access/interactions and access to/financial burden associated with laryngectomy consumables. The combined findings of this study have highlighted the care challenges that can exist for IWL. As such, healthcare providers, including SLPs, should work towards addressing these issues to ease the healthcare journey, optimise clinical outcomes, and enhance quality of life for IWL.

## Funding

The authors have nothing to report.

## Ethics Statement

Ethical approval was sought and granted by White Plains Hospital (WPH), Montefiore Medical Center/Albert Einstein College of Medicine (MMC) and The University of Queensland (UQ) (WPH & MMC Ref # 061125, IRB# #2020‐11238, UQ # 2020001304/ʼʼ#20‐030).

## Consent

Individual written consent was provided by all participants. The patient information and consent forms clearly stated that de‐identified results would be published.

## Conflicts of Interest

The authors have no conflicts of interest to declare.

## Data Availability

The dataset generated during this study is not publicly available to maintain the confidentiality of the participants and the relevant health services. Access to this data may be available at the request to the authors and relevant human research and ethics committee.

## 1

Interview guide for patient participants.

**Table:**

Questions related to all health services attended/accessed:	
1.	Tell me about the health services you have received for your laryngectomy care.
2.	What health services helped you?
3.	Where did you receive these health services?
4.	What are some of the challenges you faced in receiving/accessing these health services?
5.	Is there anything that you did not receive that you would have liked to have received (regarding your laryngectomy care)?
**Questions related to speech‐language pathology services**:	
1.	Associated with your laryngectomy, what services did you receive for your
	a. swallowing?
2.	b. voice restoration/speech?
3.	Where and how did you receive these services?
4.	What was beneficial about these services?
5.	What was challenging about accessing/receiving these services?

## References

[jlcd70326-bib-0001] Alkandari, M. , K. Ryan , and A. Hollywood . 2019. “The Experiences of People Living With Peripheral Neuropathy in Kuwait‐A Process Map of the Patient Journey.” Pharmacy 7, no. 3: 127. 10.3390/pharmacy7030127. 31480223 PMC6789644

[jlcd70326-bib-0002] Beeram, M. , A. Kennedy , and N. Hales . 2021. “Barriers to Comprehensive Multidisciplinary Head and Neck Care in a Community Oncology Practice.” *American Society of Clinical Oncology educational Book. American Society of Clinical Oncology* *Annual Meeting*, 41: 1–10. 10.1200/EDBK_320967. 34010055

[jlcd70326-bib-0003] Bickford, J. M. , J. Coveney , J. Baker , and D. Hersh . 2018. “Support Following Total Laryngectomy: Exploring the Concept From Different Perspectives.” European Journal of Cancer Care 27: e12848. https://doi‐org.ezproxy.library.uq.edu.au/10.1111/ecc.12848. 29671922 10.1111/ecc.12848

[jlcd70326-bib-0004] Braun, V. , and V. Clarke . 2019. “Reflecting on Reflexive Thematic Analysis.” Qualitative Research in Sport, Exercise and Health 11, no. 4: 589–597. 10.1080/2159676X.2019.1628806.

[jlcd70326-bib-0005] Bulto, L. N. , E. Davies , J. Kelly , and J. M. Hendriks . 2024. “Patient Journey Mapping: Emerging Methods for Understanding and Improving Patient Experiences of Health Systems and Services.” European Journal of Cardiovascular Nursing 23, no. 4: 429–433. 10.1093/eurjcn/zvae012. 38306596

[jlcd70326-bib-0006] Burns, C. L. , E. C. Ward , A. J. Hill , S. Kularatna , J. Byrnes , and L. M. Kenny . 2017. “Randomized Controlled Trial of a Multisite Speech Pathology Telepractice Service Providing Swallowing and Communication Intervention to Patients With Head and Neck Cancer: Evaluation of Service Outcomes.” Head and Neck 39, no. 5: 932–939. 10.1002/hed.24706. 28225567

[jlcd70326-bib-0007] Cancer Council Victoria and Department of Health Victoria . 2021. Optimal Care Pathway for People *With* Head and Neck *Cancer* . 2nd ed. Cancer Council Victoria.

[jlcd70326-bib-0008] Chapman, P. , K. Galvin , K. Blyth , and J. Skeat . 2026. “Navigating Life After Laryngectomy: A Qualitative Study on Adjustment and Distress in the First 6 Months Following Surgery.” Supportive Care in Cancer 34: Article130. 10.1007/s00520-024-09079-2. 41817875 PMC12982239

[jlcd70326-bib-0009] Charlton, M. , J. Schlichting , C. Chioreso , M. Ward , and P. Vikas . 2015. “Challenges of Rural Cancer Care in the United States.” Oncology 29, no. 9: 633–640. 26384798

[jlcd70326-bib-0010] Chen, A. , D. Dallender , C. Mansyur , K. Reyna , E. Limitone , and H. Geopfert . 2000. “The Impact of Clinical Pathways on the Practice of Head and Neck Oncologic Surgery.” Archives of Otolaryngology—Head and Neck Surgery 126, no. 3: 322–326. 10.1001/archotol.126.3.322. 10722004

[jlcd70326-bib-0011] Chen, M. M. , U. C. Megwalu , J. Liew , D. Sirjani , E. L. Rosenthal , and V. Divi . 2019. “Regionalization of Head and Neck Cancer Surgery May Fragment Care and Impact Overall Survival.” The Laryngoscope 129, no. 6: 1413–1419. 10.1002/lary.27440. 30152007

[jlcd70326-bib-0012] Chen, Y. J. , Y. H. Lai , Y.‐H. Lee , K. Y. Tsai , M. K. Chen , and M. Y. Hsieh . 2021. “Impact of Illness Perception, Mental Adjustment, and Sociodemographic Characteristics on Return to Work in Patients With Head and Neck Cancer.” Supportive Care in Cancer 29, no. 3: 1519–1526. 10.1007/s00520-020-05640-5. 32720008

[jlcd70326-bib-0013] Clarke, P. , K. Radford , M. Coffey , and M. Stewart . 2016. “Speech and Swallow Rehabilitation in Head and Neck Cancer: United Kingdom National Multidisciplinary Guidelines.” Journal of Laryngology & Otology 130, no. S2: S176–S180. 27841134 10.1017/S0022215116000608 PMC4873894

[jlcd70326-bib-0014] Dawson, C. , J. Graystone , K. Shah , et al. 2025. Head and Neck Cancer: GIRFT Programme National Specialty Report. NHS England.

[jlcd70326-bib-0015] DeMaddelena, H. 2002. “The Influence of Early Speech Rehabilitation With Voice Prostheses on the Psychological state of Laryngectomized Patients.” European Archives of Otorhinolaryngology 259: 48–52. 11954927 10.1007/pl00007529

[jlcd70326-bib-0016] de Souza, J. A. , S. Kung , J. O'Connor , and B. J. Yap . 2017. “Determinants of Patient‐Centered Financial Stress in Patients With Locally Advanced Head and Neck Cancer.” Journal of Oncology Practice 13, no. 4: e310–e318. 10.1200/JOP.2016.016337. 28195811

[jlcd70326-bib-0017] Dooks, P. , M. McQuestion , D. Goldstein , and A. Molassiotis . 2012. “Experiences of Patients With Laryngectomy as They Reintegrate Into Their Community.” Supportive Care Cancer 20: 489–498. 10.1007/s00520-011-1101-4 21298450

[jlcd70326-bib-0018] Ewers, C. , J. M. Patterson , and L.‐J. Watson . 2023. “Patient Experience of Telehealth Appointments in Head and Neck Cancer Services During the COVID‐19 Pandemic.” *International Journal of Language & Communication Disorders* 59, no. 3: 991–1001. 10.1111/1460-6984.12974. 37929624

[jlcd70326-bib-0019] Farrugia, M. , H. Yu , S. J. Ma , et al. 2021. “Financial Counselling Is Associated With Reduced Financial Difficulty Scores in Head and Neck Cancer Patients Treated With Radiation Therapy.” Cancers 13, no. 11: 2516. 10.3390/cancers13112516. 34063890 PMC8196601

[jlcd70326-bib-0020] Foley, J. , C. L. Burns , E. C. Ward , et al. 2022. “Post‐Acute Health Care Needs of People With Head and Neck Cancer: Mapping Health Care Services, Experiences, and the Impact of Rurality.” Head and Neck 44, no. 6: 1377–1392. 10.1002/hed.27037. 35319137 PMC9313784

[jlcd70326-bib-0021] Foley, J. , E. C. Ward , C. L. Burns , et al. 2023. “Enhancing Speech‐Language Pathology Head and Neck Cancer Service Provision in Rural Australia: Using a Plan, Do, Study, Act Approach.” International Journal of Speech‐Language Pathology 25, no. 2: 292–305. 10.1080/17549507.2022.2050300. 35532005

[jlcd70326-bib-0022] Garstka, M. , D. Monlezun , and E. Kandil . 2020. “Does Distance to Treatment Affect Mortality Rate for Surgical Oncology Patients?” American Surgeon 86, no. 9: 1129–1134. 10.1177/0003134820943649. 32955355

[jlcd70326-bib-0023] Goepfert, R. P. , K. A. Hutcheson , J. S. Lewin , et al. 2017. “Complications, Hospital Length of Stay, and Readmission After Total Laryngectomy.” Cancer 123, no. 10: 1760–1767. 10.1002/cncr.30483. 28026864 PMC5862421

[jlcd70326-bib-0024] Graboyes, E. M. , M. Chappell , K. A. Duckett , et al. 2023. “Patient Navigation for Timely, Guideline‐Adherent Adjuvant Therapy for Head and Neck Cancer: A National Landscape Analysis.” Journal of the National Comprehensive Cancer Network: JNCCN 21, no. 12: 1251–1259.e5. 10.6004/jnccn.2023.7061. 38081134 PMC10846494

[jlcd70326-bib-0025] Graboyes, E. M. , D. Kallogjeri , M. J. Saeed , M. A. Olsen , and B. Nussenbaum . 2017. “Postoperative Care Fragmentation and Thirty‐Day Unplanned Readmissions after Head and Neck Cancer Surgery.” Laryngoscope 127, no. 4: 868–874. 10.1002/lary.26301. 27740687 PMC5493141

[jlcd70326-bib-0026] Graneheim, U. H. , and B. Lundman . 2004. “Qualitative Content Analysis in Nursing Research: Concepts, Procedures and Measures to Achieve Trustworthiness.” Nurse Education Today 24, no. 2: 105–112. 10.1016/j.nedt.2003.10.001. 14769454

[jlcd70326-bib-0027] Hancock, K. L. , E. C. Ward , and A. E. Hill . 2020. “Speech and Language Therapists' Reflections on Developing and Maintaining Confidence in Tracheoesophageal Speech Rehabilitation.” International Journal of Language and Communication Disorders 55, no. 1: 85–96. 10.1111/1460-6984.12505. 31612612

[jlcd70326-bib-0028] Hanna, E. , S. Schultz D. Doctor , E. Vural , S. Stern , and J. Suen . 1999. “Development and Implementation of a Clinical Pathway for Patients Undergoing Total Laryngectomy: Impact on Cost and Quality of Care.” Archives of Otolaryngology Head and Neck Surgery 125, no. 11: 1247–1251. 10.1001/archotol.125.11.1247. 10555697

[jlcd70326-bib-0029] Hinni, M. L. , and L. R. Crujido . 2013. “Laryngectomy Rehabilitation: A Perspective From the United States of America.” Current Opinion in Otolaryngology and Head and Neck Surgery 21, no. 3: 218–223. 10.1097/MOO.0b013e3283604001. 23511606

[jlcd70326-bib-0030] Homer, J. J. , S. C. Winter , E. C. Abbey , et al. 2024. “Head and Neck Cancer: United Kingdom National Multidisciplinary Guidelines, Sixth Edition.” The Journal of laryngology and otology 138, no. S1: S1–S224. 10.1017/S0022215123001615. 38482835

[jlcd70326-bib-0031] Jacobson, J. J. , J. B. Epstein , F. C. Eichmiller , et al. 2012. “The Cost Burden of Oral, Oral Pharyngeal, and Salivary Gland Cancers in Three Groups: Commercial Insurance, Medicare, and Medicaid.” Head and Neck Oncology 4: 15. 10.1186/1758-3284-4-15. 22537712 PMC3503554

[jlcd70326-bib-0032] Johnson, S. , J. T. McDonald , and M. J. Corsten . 2008. “Socioeconomic Factors in Head and Neck Cancer.” Journal of Otolaryngology—Head and Neck Surgery 37, no. 4: 597–601. 19128600

[jlcd70326-bib-0033] Kagan, S. H. , B. Morgan , T. Smink , et al. 2020. “The Oncology Nurse Navigator as “Gate Opener” to Interdisciplinary Supportive and Palliative Care for People With Head and Neck Cancer.” Journal of oncology navigation & survivorship 11, no. 8: 259–266. 32775043 PMC7409946

[jlcd70326-bib-0034] Kelly, J. , J. Dwyer , T. Mackean , K. O. Donnell , and E. Willis . 2017. “Coproducing Aboriginal Patient Journey Mapping Tools for Improved Quality and Coordination of Care.” Australian Journal of Primary Health 23, no. 6: 536–542. 10.1071/PY16069. 27927279

[jlcd70326-bib-0035] Kushniruk, A. W. , E. Borycki , and A. Parush . 2020. “A Case Study of Patient Journey Mapping to Identify Gaps in Healthcare: Learning From Experience With Cancer Diagnosis and Treatment.” Knowledge Management & E‐Learning: An International Journal 12: 405–418. 10.34105/j.kmel.2020.12.022.

[jlcd70326-bib-0036] Levin, R. , R. Ferraro , S. Kodosky , and F. Fedok . 2000. “The Effectiveness of a “Critical Pathway” in the Management of Laryngectomy Patients.” Head and Neck 22, no. 7: 694–699. 10.1002/1097-0347(200010)22:7<694::aid-hed9>3.0.co;2-0. 11002325

[jlcd70326-bib-0037] Lewis, C. M. , Z. Nurgalieva , E. M. Sturgis , S. Y. Lai , and R. S. Weber . 2016. “Improving Patient Outcomes Through Multidisciplinary Treatment Planning Conference.” Head and Neck 38, no. S1: E1820–E1825. 10.1002/hed.24325. 26690552

[jlcd70326-bib-0038] Licitra, L. , U. Keilholz , M. Tahara , et al. 2016. “Evaluation of the Benefit and Use of Multidisciplinary Teams in the Treatment of Head and Neck Cancer.” Oral Oncology 59: 73–79. 10.1016/j.oraloncology.2016.06.002. 27424185

[jlcd70326-bib-0039] Longobardi, Y. , V. Savoia , C. Parilla , et al. 2023. “Pre‐Operative Speech‐Language Pathology Counselling in Patients Undergoing Total Laryngectomy: A Pilot Randomized Clinical Trial.” Current Psychology 42, no. 7: 5717–5727. 10.1007/s12144-021-01932-z.

[jlcd70326-bib-0040] Ly, S. , F. Runacres , and P. Poon . 2021. “Journey Mapping as a Novel Approach to Healthcare: A Qualitative Mixed Methods Study in Palliative Care.” BMC Health Services Research 21, no. 1: 915. 10.1186/s12913-021-06934-y. 34479541 PMC8417950

[jlcd70326-bib-0041] Maddox, P. T. , and L. Davies . 2012. “Trends in Total Laryngectomy in the Era of Organ Preservation.” Otolaryngology—Head and Neck Surgery 147, no. 1: 85–90. 10.1177/0194599812438170. 22371344

[jlcd70326-bib-0042] Malterud, K. , V. D. Siersma , and A. D. Guassora . 2016. “Sample Size in Qualitative Interview Studies: Guided by Information Power.” Qualitative Health Research 26, no. 13: 1753–1760. 10.1177/1049732315617444. 26613970

[jlcd70326-bib-0070] Massa, S. T. , N. Osazuwa‐Peters , E. Adjei Boakye , R. J. Walker , and G. M. Ward . 2019. “Comparison of the Financial Burden of Survivors of Head and Neck Cancer With Other Cancer Survivors.” JAMA Otolaryngology‐Head & Neck Surgery 145, no. 3: 239–249. 10.1001/jamaoto.2018.3982. 30789634 PMC6439752

[jlcd70326-bib-0043] Massa, S. T. , S. Chidambaram , P. Luong , E. M. Graboyes , and A. L. Mazul . 2022. “Quantifying Total and Out‐of‐Pocket Costs Associated With Head and Neck Cancer Survivorship.” JAMA Otolaryngology—Head and Neck Surgery 148, no. 12: 1111. 10.1001/jamaoto.2022.3269. 36264567 PMC9585466

[jlcd70326-bib-0044] Massa, S. T. , A. P. Liebendorfer , J. P. Zevallos , and A. L. Mazul . 2020. “Distance Traveled to Head and Neck Cancer Provider: A Measure of Socioeconomic Status and Access.” Otolaryngology—Head and Neck Surgery 162, no. 2: 193–203. 10.1177/0194599819892015. 31794337

[jlcd70326-bib-0045] Messing, B. P. , E. C. Ward , C. Lazarus , et al. 2019. “Establishing a Multidisciplinary Head and Neck Clinical Pathway: an Implementation Evaluation and Audit of Dysphagia‐Related Services and Outcomes.” Dysphagia 34, no. 1: 89–104. 10.1007/s00455-018-9917-4. 29922848 PMC6349813

[jlcd70326-bib-0046] Meulemans, J. , H. Demarsin , J. Debacker , et al. 2020. “Functional Outcomes and Complications After Salvage Total Laryngectomy for Residual, Recurrent, and Second Primary Squamous Cell Carcinoma of the Larynx and Hypopharynx: A Multicenter Retrospective Cohort Study.” Frontiers in Oncology 10: 1390. 10.3389/fonc.2020.01390. 32983968 PMC7492266

[jlcd70326-bib-0047] Meyer, M. 2019. “Mapping the Patient Journey across One Continuum: Lessons Learned From One Patient's Experience.” Journey of Patient Experience 6, no. 2: 103–107. https://doi.org10.1177/2374373518783763. 10.1177/2374373518783763 PMC6558942 31218254

[jlcd70326-bib-0048] Middleton, S. , J. Barnett , and D. Reeves . 2001. “What is an integrated care pathway?.” www.evidence‐based‐medicine.co.uk.

[jlcd70326-bib-0049] Miller, A. , E. Wilson , and C. Diver . 2023. “Returning to Work: A Qualitative Study of the Experiences of Head and Neck Cancer Survivors.” Journal of Laryngology and Otology 1–20. 10.1017/S0022215122002201. 36999532

[jlcd70326-bib-0050] Mott, N. M. , M. L. Mierzwa , K. A. Casper , et al. 2022. “Financial Hardship in Patients With Head and Neck Cancer.” JCO Oncology Practice 18, no. 6: e925–e937. 10.1200/OP.21.00683. 35167324 PMC9797234

[jlcd70326-bib-0051] National Comprehensive Cancer Network . 2023. “NCCN Clinical Practice Guidelines in Oncology: Head and Neck Cancers.” NCCN Clinical Practice Guidelines in Oncology. Version 2.2023.

[jlcd70326-bib-0052] Rourke, E. J. 2021. “Continuity, Fragmentation, and Adam Smith.” New England Journal of Medicine 385, no. 19: 1810–1814. 10.1056/NEJMms2103844. 34731542

[jlcd70326-bib-0053] Saade, R. , C. Jabbour , E. Keyrouz , et al. 2025. “Survival and Larynx Function After Upfront vs. Salvage Total Laryngectomy: A Meta‐Analysis.” *European Archives of Oto‐Rhino‐Laryngology*. 10.1007/s00405-025-09647-8. Epub ahead of print. 40968204

[jlcd70326-bib-0054] Saragosa, M. , S. Nizzer , S. McKay , and K. Kuluski . 2023. “The Hospital‐to‐Home Care Transition Experience of Home Care Clients: an Exploratory Study Using Patient Journey Mapping.” BMC Health Services Research 23, no. 1: 934. 10.1186/s12913-023-09899-2. 37653515 PMC10469468

[jlcd70326-bib-0055] Saraswathula, A. , J. M. Austin , C. Fakhry , et al. 2023. “Surgeon Volume and Laryngectomy Outcomes.” Laryngoscope 133, no. 4: 834–840. 10.1002/lary.30229. 35634691 PMC9708934

[jlcd70326-bib-0056] Stanisce, L. , M. McGlone , Y. Koshkareva , et al. 2023. “Socioeconomic Influence on Speech Rehabilitation After Total Laryngectomy.” Otolaryngology—Head and Neck Surgery 169, no. 6: 1499–1505. 10.1002/ohn.412. 37422889

[jlcd70326-bib-0057] Swann, J. 2017. “Process Mapping: Looking at the Patient's Full Journey.” British Journal of Healthcare Assistants 11, no. 4: 196–200. 10.12968/bjha.2017.11.4.196.

[jlcd70326-bib-0058] Tong, A. , P. Sainsbury , and J. Craig . 2007. “Consolidated Criteria for Reporting Qualitative Research (COREQ): A 32‐Item Checklist for Interviews and Focus Groups.” International Journal for Quality in Health Care 19, no. 6: 349–357. 10.1093/intqhc/mzm042. 17872937

[jlcd70326-bib-0059] Trebble, T. M. , N. Hansi , T. Hydes , M. A. Smith , and M. Baker . 2010. “Process Mapping the Patient Journey: An Introduction.” BMJ 341: c4078. 10.1136/bmj.c4078. 20709715

[jlcd70326-bib-0060] van Sluis, K. E. , A. F. Kornman , L. van der Molen , M. W. M. van den Brekel , and G. Yaron . 2020. “Women's Perspective on Life after Total Laryngectomy: A Qualitative Study.” International Journal of Language & Communication Disorders 55, no. 2: 188–199. 10.1111/1460-6984.12511. 31674722 PMC7079180

[jlcd70326-bib-0061] van Walraven, C. , N. Oake , A. Jennings , and A. J. Forster . 2010. “The Association Between Continuity of Care and Outcomes: A Systematic and Critical Review.” Journal of Evaluation in Clinical Practice 16, no. 5: 947–956. 10.1111/j.1365-2753.2009.01235.x. 20553366

[jlcd70326-bib-0062] Verdonck‐de Leeuw, I. M. , C. Dawson , L. Licitra , et al. 2022. “European Head and Neck Society Recommendations for Head and Neck Cancer Survivorship Care.” Oral Oncology 133: 106047. 10.1016/j.oraloncology.2022.106047. 35932637

[jlcd70326-bib-0063] Ward, E. C. , R. Burnett , A. Morton , et al. 2007. “Management of Head and Neck Cancer: An International Perspective.” In Head and Neck Cancer: Treatment, Rehabilitation and Outcomes, edited by E. C. Ward and C. Van As‐Brooks , 381–400. Plural Publishing.

[jlcd70326-bib-0064] Watson, L.‐J. , E. Fahy , D. W. Hamilton , L. Sharp , V. Thornton , and J. M. Patterson . 2025. “Laryngectomy Education and Training Resources: An Environmental Scan of Resources.” *Journal of Cancer Education*. Advance online publication. 10.1007/s13187-025-02606-1. PMC13157435 40500579

[jlcd70326-bib-0065] Watson, L. J. , D. Hamilton , and J. M. Patterson . 2022. “Patient Experience of the Acute Post‐Surgical Period Following Total Laryngectomy during the COVID‐19 Era.” International Journal of Language and Communication Disorders 57, no. 4: 737–748. 10.1111/1460-6984.12709. 35403774 PMC9111097

